# An evidence-based approach to the development and implementation of a podiatric rheumatology service within the New Zealand health sector

**DOI:** 10.1186/1757-1146-4-S1-O40

**Published:** 2011-05-20

**Authors:** Keith Rome, Peter Gow, Nicola Dalbeth

**Affiliations:** 1 AUT University, Health & Rehabilitation Research Institute, Auckland, New Zealand; 2 Counties Manakau District Health Board, Auckland, New Zealand; 3 Auckland District Health Board, Auckland, New Zealand; 4 University of Auckland, Auckland, New Zealand

## 

Foot problems are extremely common in patients with rheumatoid arthritis (RA) and gout. There is ample evidence that foot pain, either alone or as a co-morbidity, contributes significantly to disability. Despite the high prevalence of foot disease, the problem is often trivialised or underappreciated. The inequity in foot health provision for patients with rheumatic disorders in New Zealand has recently been highlighted. Expertise in dealing with foot problems is often limited among healthcare professionals, and it has been argued that better integration of podiatric services into rheumatology services would be beneficial. The evidence base for dedicated podiatry as part of multidisciplinary foot clinics in diabetes is well established but this has yet to be achieved for rheumatology services. However, the role of the podiatrist in the rheumatology team is becoming recognised as a vital component in the integrated care given to patients by the multidisciplinary team. The goal of the podiatry element of rheumatology care is to reduce foot-related pain, maintain/improve foot function and so mobility, while protecting skin and other tissues from damage. The aim of this presentation is to highlight the major issues related to foot care for patients with arthritis and provide key recommendations that should implemented to improve access to podiatric services in New Zealand. The presentation will also provide evidence from recent studies conducted by the research team to support the justification of a podiatric rheumatology service.

